# Do personality profiles contribute to patterns of physical activity and sedentary behavior in adulthood? A prospective cohort study

**DOI:** 10.1186/s12966-024-01662-y

**Published:** 2024-09-26

**Authors:** Johanna Ahola, Tiia Kekäläinen, Sebastien Chastin, Timo Rantalainen, Marja-Liisa Kinnunen, Lea Pulkkinen, Katja Kokko

**Affiliations:** 1 Gerontology Research Center and Faculty of Sport and Health Sciences, University of Jyväskylä, Jyväskylä, Finland; 2 School of Health and Life Sciences, Glasgow Caledonian University, Glasgow, UK; 3 Department of Movement and Sports Sciences, Ghent University, Ghent, Belgium; 4 The Wellbeing Services County of Central Finland, Jyväskylä, Finland; 5 School of Medicine, University of Eastern Finland, Kuopio, Finland; 6 Department of Psychology, University of Jyväskylä, Jyväskylä, Finland

**Keywords:** Personality trait, Bout analysis, Accelerometer

## Abstract

**Background:**

Despite the observed associations of personality traits with levels of moderate-to-vigorous physical activity (MVPA) and sedentary behavior (SB), studies exploring whether the personality profiles differ in terms of the pattern of accumulation of physical behavior are lacking. The aim of this study was to identify adults’ personality profiles and to characterize and investigate how these profiles differ in physical behavior.

**Methods:**

The study utilized the longitudinal data of the participants of the Jyväskylä Longitudinal Study of Personality and Social Development (*n* = 141–307). Information on the five-factor model of personality, including the traits of neuroticism, extraversion, conscientiousness, openness, and agreeableness, was collected at ages 33, 42, 50, and 61 years, and used to create latent personality profiles. Physical behavior, operationalized as the amount and accumulation of MVPA and SB bouts, was captured using a triaxial accelerometer worn during waking hours at age 61 years. The differences in the behavior between the personality profiles were analyzed using the Kruskal-Wallis test.

**Results:**

Five personality profiles were identified: *resilient* (20.2%), *brittle* (14.0%), *overcontrolled* (9.8%), *undercontrolled* (15.3%), and *ordinary* (40.7%). Although there were no statistically significant differences between the personality profiles in the time spent in MVPA relative to SB (MVPA per hour of daily SB), individuals with *resilient* (low in neuroticism and high in other traits) and *ordinary* (average in each trait) profiles had MVPA-to-SB ratios of 0.12 (7 min) and those with a *brittle* (high in neuroticism and low in extraversion) profile had a ratio of 0.09 (5.5 min). The individuals in the *resilient* group exhibited a longer usual MVPA bout duration than those in the *overcontrolled* (low in extraversion, openness, and agreeableness) (8 min vs. 2 min) and *undercontrolled* (high in openness and low in conscientiousness) groups (8 min vs. 3 min). They also exhibited a longer usual SB bout duration than those in the *ordinary* group (29 min vs. 23 min).

**Conclusions:**

The *resilient* group displayed the most prolonged MVPA and SB bout patterns. The results suggest that personality characteristics may contribute to how MVPA and SB are accumulated.

**Supplementary Information:**

The online version contains supplementary material available at 10.1186/s12966-024-01662-y.

## Background

Promoting physical activity (PA) and limiting sedentary behavior (SB) are key factors in solving the current public health issues. In addition to the amounts of these behaviors being independently linked to many health outcomes [1], they are also jointly associated with mortality risk [2, 3]. The 10-year mortality risk among 50- to 79-year-old participants decreased more sharply after the proportion of moderate-to-vigorous physical activity (MVPA) per time spent sedentary exceeded one-tenth [2]. Thus, replacing SB with MVPA is recommended. Recent evidence also suggests that MVPA bouts of any length should be counted for health benefits (e.g., lower mortality risk) [1, 4]. However, the effects of SB on health may be bout duration dependent, with continuous SB bouts of less than 30 min being physiologically less harmful (e.g., lower blood pressure and mortality risk) than bouts longer than 30 min [5, 6]. Given the potential public health relevance, it is important to examine multidimensional PA and SB, such as the manner in which they accrue while awake [7].

The personality traits that form the basic layer of the three layers of personality [8] have consistently been associated with total amounts of PA, particularly MVPA and SB [9–13]. There is a consensus among the trait theories that five factors capture the majority of the relatively stable individual differences in the ways in which people think, feel, and behave [14]. This so-called five-factor model postulates that the five traits are neuroticism, extraversion, conscientiousness, openness, and agreeableness [15], which are commonly measured using the NEO Five-Factor Inventory (NEO-FFI) [16]. Of these traits, the strongest correlates of PA and SB are *conscientiousness*, which showed a positive correlation with PA and negative correlation with SB; *neuroticism*, which showed a positive correlation with SB and negative correlation with PA; and *extraversion*, which showed a positive correlation only with PA [9–13]. These traits are followed by *openness*, which showed a weaker positive correlation with PA [9, 12], and *agreeableness*, which has not been significantly associated with PA or SB [9–13].

There is a need to go beyond single traits to improve the ecological validity of the research, as in real life, a person expresses various personality traits simultaneously [17]. Personality traits may function together [17] and modify each other’s associations with multiple outcomes. For instance, individuals with high levels of neuroticism and low conscientiousness showed a negative correlation with PA, whereas those characterized by high neuroticism combined with high conscientiousness did not have such a relationship [18].

A few studies have identified four or five combinations of the five personality traits, that is, personality profiles based on latent profile analysis (LPA) and applied them to the context of health [19, 20] or health-related behaviors [21]. Personality profiles, whose names have been heterogenous, can be outlined through the framework of activity and self-regulation, the latter including components of both emotion regulation and behavior regulation [17]. Compared with individuals with other profiles, those characterized as high in all traits except neuroticism and thus high in both activity and emotional self-regulation (called *resilient*) reported better self-rated health, fewer psychosomatic symptoms, and lower psychological distress in the Jyväskylä Longitudinal Study of Personality and Social Development (JYLS) [20]. That longitudinal study examined the same cohort as in the present study until the age of 50 years. In other studies, individuals with a *resilient* profile reported better physical health [19] and more frequent engagement in moderate PA during leisure time [21]. In contrast to the *resilient* profile, people high in neuroticism but low in all other traits and thus low in both activity and emotional self-regulation (previously called *overcontrolled* [20] but renamed *brittle* in the same longitudinal study after considering the two components of self-regulation [17]; called *overcontroller* [19]) rated their health worse and experienced more psychosomatic symptoms, higher psychological distress [20], and poorer physical and mental health [19] than those with other profiles. In the JYLS, the three other profiles were, first, low in activity and high in behavioral self-regulation (previously called *reserved* but renamed *overcontrolled* in the same longitudinal study after considering the two components of self-regulation [17]); second, high in activity and low in behavioral self-regulation (called *undercontrolled*); and third, average in both activity and self-regulation (called *ordinary*) [20]. Evaluations of the subjective health of these three groups lay between the *resilient* and *brittle* profiles, while no associations were found between the personality profiles and objective indicators of health, such as body mass index (BMI) and blood pressure [20]. Apart from a study that compared the *resilient* profile to the other profiles [21], the role of personality profiles in physical behavior remains unknown.

As previous research has predominantly focused on investigating the relevance of single personality traits in relation to self-reported levels of MVPA and SB [9], studies that explored whether personality profiles differ in terms of the pattern of accumulation of measured physical behavior are lacking (Table 1). It is important to examine device-assessed physical behavior, as the associations of personality characteristics with the device-based measures of MVPA and SB differ from self-reports [22]. Device-based measures enable the capture of the entire period that they are worn and thus the exploration of the pattern of accumulation of behavior using metrics such as intensity, frequency, and duration [23]. These characteristics make them particularly suitable for investigating also other aspects of multidimensional physical behavior, such as SB [24], which is more difficult to assess with self-reports and has been less covered in personality research than PA.

**Table 1:** Summary of the existing knowledge on the associations of personality traits and profiles with PA and SB, and new contributions of the present study

The existing knowledge					New contributions of the present study
Personality trait		Description^a^	PA^b^	SB^b^	
	neuroticism	tendency to be anxious and unstable	–	+	Novel insights into personality profiles relevant for PA and SB including
	conscientiousness	tendency to be organized and responsible	+	–	
	extraversion	tendency to be outgoing and active	+	na	– a set of personality profiles with better ecological validity than single personality traits
	openness	tendency to be curious and imaginative	+	na	
	agreeableness	tendency to be kind and trusting	na	na	– a detailed device-based analysis of multidimensional PA and SB
Personality profile		Description^c^	PA^c^	SB	
	resilient (vs. overcontrolled, undercontrolled and average profiles)	a combination of low neuroticism and high other four traits	+	?	

Note. – = a negative correlation, + = a positive correlation, na = no association, ? = not studied

^a^ [15], ^b^ [9–13], ^c^ [21]

The aim of this study was to identify adults’ personality profiles based on the NEO-FFI and to characterize and investigate how these profiles differ in physical behavior. Specifically, we aimed to investigate whether the profiles differ in multiple metrics of accelerometer-measured physical behavior, namely (1) the ratio of MVPA to SB, and (2) their accumulation patterns described as usual bout durations [25]. As the *resilient* and *brittle* profiles represented the extremes of subjective health in previous publication based on the same longitudinal study [20], they were hypothesized to differ in physical behavior similarly.

## Methods

### Study design and participants

The study used longitudinal data from an ongoing cohort study, the JYLS [17, 26]. The JYLS was launched in 1968 when participants from 12 randomly selected and complete second-grade school classes in Jyväskylä, Central Finland were recruited. The recruitment process resulted in a representative sample of 369 participants (53% males, native Finns, mainly born in 1959) with no initial attrition. Since age 8 years, the same participants have been followed in major waves at ages 14, 27, 36, 42, 50 [17], and 61 years [26]. The participants were also approached at age 33 years to participate in, for example, personality measurement. Throughout the adult years, the data collection methods have included life situation questionnaires, interviews, inventories, health examinations, and PA monitoring [17, 26].

The data used in the present study were collected at ages 33 (1992), 42 (2001), 50 (2009), and 61 years (2020–2021), with emphasis on the most recent data collection that involved device-based measurement of physical behavior (TRAILS, Transitions at Age 60: Individuals Navigating Across the Lifespan) [26]. The remaining study sample in adulthood has reasonably well represented both the initial sample and the same-age Finnish cohort on several sociodemographic characteristics [17, 26]. More information on the recruitment and representativeness of the sample is documented in the Supplementary Material (Additional File 1). The analytical sample in this study consisted of 141–307 participants, depending on the analysis in question. Both personality and accelerometer data were available for 141 participants.

The data acquisition was conducted in accordance with the Declaration of Helsinki. The procedures were approved by the ethics committee of either the Central Finland Health District (ages 42 years [no. 42/2000] and 50 years [no. 10E/2008]) [27, 28] or the University of Jyväskylä (age 61 years [December 13, 2019]) [26], depending on the prevailing requirements concerning data collection. The adult-aged participants also signed a written informed consent form each time to confirm their voluntary participation [17, 26].

### Measures

The NEO-FFI was used to assess *personality traits* at ages 33, 42, 50, and 61 years [16]. Among the 60 common items repeated at each age, 12 items measured each of the five higher-order personality traits: *neuroticism*, *extraversion*, *openness*, *agreeableness*, and *conscientiousness*. The participants rated the items on a 5-point Likert-type scale ranging from *1 = strongly disagree* to *5 = strongly agree*. Means were calculated. Both the original scale and the Finnish translation have shown a clear five-factor structure and good internal consistency, indicating that similar personality traits can be identified across cultures with the current measurement tool [29–31]. In the present study, the Cronbach alpha values indicated the high internal consistency of the scales, ranging from 0.73 to 0.88 (ages 33–50 years reported by Kinnunen et al. [20]). Data were obtained from 307 participants who completed the personality inventory four (*n* = 127), three (*n* = 66), two (*n* = 47), or one time (*n* = 67).

A triaxial accelerometer (sampling rate of 100 Hz, units of gravity [*g*], UKK RM42, UKK Terveyspalvelut Oy, Tampere, Finland) was used to monitor physical behavior, namely *MVPA* and *SB*, at age 61 years. Data were collected between March 2020 and May 2021, excluding the spring 2020 state of emergency due to COVID-19 (March 16, 2020–June 16, 2020). During the 1-year measurement period, Finland had mild restrictions and favorable outdoor PA opportunities for 60-year-olds [32]. Those interested in health examinations (*n* = 179) were offered with and instructed on how to use hip-placed monitors. Participants who were willing to participate were asked to wear the monitor during waking hours for 7 consecutive days and remove it only during water-related activities and sauna. In addition, they were asked to provide information on their in-bed and out-of-bed times, times of starting and finishing work, non-step-based activities (e.g., cycling and swimming), and periods when the accelerometer was removed for longer than 30 min, on a diary.

Raw acceleration data were used to calculate the mean amplitude deviation (MAD, g) [33]. Non-overlapping epoch lengths of 5 s were summed up to an average of 60-s MADs using a custom-written script on MATLAB (version R2016b; The MathWorks Inc., Natick MA, USA). Data were classified according to thresholds of ≥ 0.091 g for MVPA and < 0.0167 g for SB [33, 34]. The hip-worn device did not detect body posture; thus, in addition to the consensus definition of SB, including low energy consuming behaviors performed in a seated, reclined, or lying posture [7], the used variable also captured standing without ambulation. A non-wear time was defined as ≥ 120 min of continuous MAD values < 0.02 g, which corresponded best to the diary-reported wear times.

As valid data requirements were set at a minimum of 4 days with at least 10 h of wear time [35], data were obtained for 142 participants. *The ratio of MVPA to SB* was calculated by dividing the individual’s mean daily MVPA by the individual’s mean daily SB (number of minutes per day during which the MAD value meets the intensity category of MVPA or SB). MVPA and SB bouts of any length were defined as a period of uninterrupted time spent on the specified signal intensity [7]. Minute-to-minute patterns of accumulation were described with the *usual bout duration* (*W*_50%_) [25] using a bespoke MATLAB script. This weighted median bout duration was chosen over the mean bout length because it is less subject to a high number of short bouts due to power-law distribution [36]. The variable describes the cumulative distribution of the bouts, indicating a level where half of the total amount of MVPA or SB is accumulated above and below (sum[probability × length] = 0.5), with higher values indicating accumulation in longer bouts [25]. The *daily amount of MVPA and SB* and the *number of MVPA and SB bouts* (number of bouts/valid days) were also documented for additional information.

The categorical background variables included *gender* (1 = women vs. 2 = men), *educational status* (1 = vocational school at most vs. 2 = vocational college or polytechnic, university), *occupational status* (1 = blue-collar worker vs. 2 = white-collar worker), and *self-rated health* (1 = fairly or very good vs. 2 = average, fairly, or extremely poor).

### Statistical analyses

LPA was used to extract subgroups of adults with respect to personality traits. We reanalyzed the personality profiles presented by Kinnunen et al. [20] (ages 33, 42, and 50 years, *n* = 304) using information also from the most recent JYLS measurement point at age 61 (ages 33, 42, 50 and 61 years, *n* = 307). Kinnunen et al. [20] found five profiles: *resilient* (*n* = 65, 21.4% of the sample), *brittle* (*n* = 40, 13.2%), *overcontrolled* (*n* = 25, 8.2%), *undercontrolled* (*n* = 41, 13.5%), and *ordinary* (*n* = 133, 43.8%). *Brittle* was labeled as *overcontrolled*, and *overcontrolled* was labeled as *reserved* in a previous study [20] (see reasons for relabeling [17]).

The LPA was performed on the Mplus version 8.8 statistical software [37] by producing models from one profile solution to eight profile solution. The indicator variables included scores for the five personality traits at each of the four measurement points. The estimation method was full information maximum likelihood with robust standard errors (MLR), which utilized information from all available data points. The starting values were set to 500. The optimal number of profiles was selected on the basis of not only the goodness-of-fit statistics (Bayesian information criterion [BIC], sample-size adjusted BIC [SABIC], Akaike information criterion [AIC], log likelihood values [LogL], entropy, adjusted Lo-Mendell-Rubin likelihood ratio test [LMR], and bootstrapped likelihood ratio test [BLRT]) but also the interpretability of the profiles [38, 39]. The sizes of the profiles were also considered. After the selection of the most suitable solution, profile membership was treated as known.

The rest of the statistical analyses were conducted using the IBM SPSS version 28 statistical software (IBM Corp., Armonk, NY, USA). Attrition in the main analysis of the study (*n* = 141) was analyzed by comparing those who provided and those who did not provide valid accelerometer data in terms of personality traits and several sociodemographic and health-related variables. The personality profiles of the participants with valid accelerometer data (*n* = 141) were also described with sociodemographic and health-related variables. Differences in background variables between the participants and non-participants, and between the profiles were tested either with a chi-square test combined with adjusted standardized residuals (statistical significance set to > 1.96) or independent samples *t*-tests.

The accelerometer data were examined visually by viewing heat maps that described the intensity spectrum over time. Data visualization, that is, plotting of the ratio of MVPA to SB and the probability distributions of MVPA and SB bouts per bout length, were performed in the R environment (Posit Software, PBC, Boston, MA, USA) [40]. Multiple metrics of physical behavior were used as the primary outcomes. To test whether the personality profiles differed in these metrics, a nonparametric Kruskal-Wallis test was used owing to the positively skewed distributions in multiple variables and limited sample size (statistical significance set to *p* < 0.05; results of the analysis of variance are reported in Additional File 2, Table S4). According to a sensitivity power analysis computed by G*Power v. 3.1.9.7 [41], the sample size of 141 participants enabled the detection of effect sizes of 0.30 or larger, with 80% power and a probability level (alpha) of 0.05. In additional analyses, the associations of single personality traits with the metrics of physical behavior were assessed with Spearman correlations. How missing information was handled is described in Supplementary Material (Additional File 1).

## Results

### Identification of personality profiles

Five personality profiles were identified (BIC = 6513.5; AIC = 6051.4). Although the six- and seven-profile solutions had better goodness-of-fit indices (6 classes: BIC = 6493.4, AIC = 5953.0; 7 classes: BIC = 6485.2, AIC = 5866.5) than the five-profile solution, the five-profile solution was chosen because of small improvements in fit indices, relatively high classification certainty (ranging from 0.85 to 0.91), and meaningfulness of the profiles. The model-fit statistics for the LPA are presented in Supplementary Material (Additional File 2, Table S3).

**Fig. 1:**
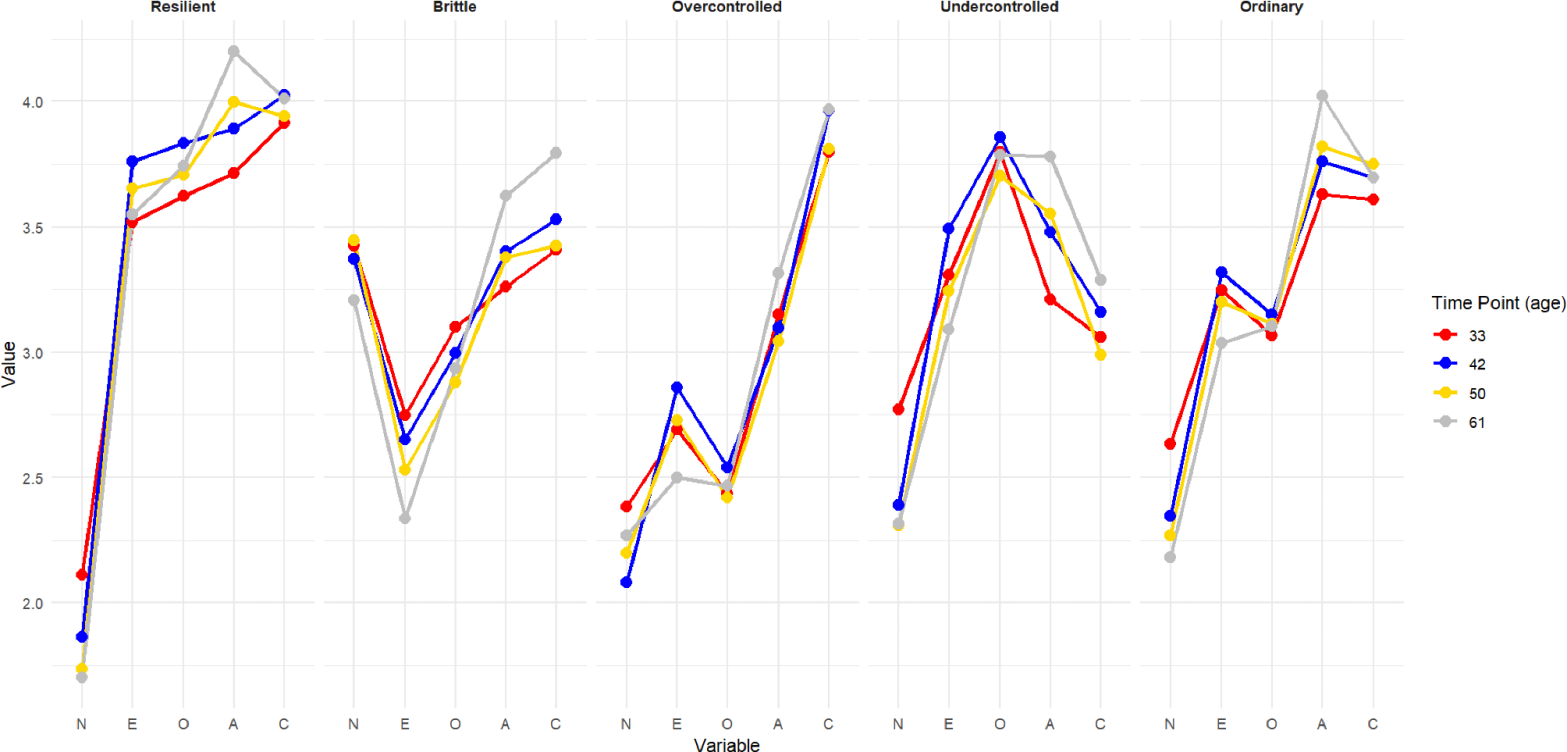
Five personality profiles at ages 33, 42, 50, and 61 years (*n* = 307; N = neuroticism, E = extraversion, O = openness, A = agreeableness, C = conscientiousness; scale 1–5)

The mean scores of the personality traits in each profile and at each measurement point over the 28-year follow-up (ages 33, 42, 50, and 61 years) are represented in Fig. 1 (*n* = 307). When the means of the profiles (traits averaged across the measurement points) were compared with the means of the whole sample (differences > 0.3 reported), the individuals with a *resilient* profile (*n* = 62, 20.2% of the sample) scored low in neuroticism and high in other traits. By contrast, those with a *brittle* profile (*n* = 43, 14.0%) scored high in neuroticism and low in extraversion. The individuals assigned to the *overcontrolled* profile (*n* = 30, 9.8%) were characterized as low in extraversion, openness, and agreeableness. On the other hand, the individuals with an *undercontrolled* profile (*n* = 47, 15.3%) scored high in openness and low in conscientiousness. The *ordinary* profile (*n* = 125, 40.7%) included the largest group of people, scoring close to the average in all five personality traits.

**Fig. 2:**
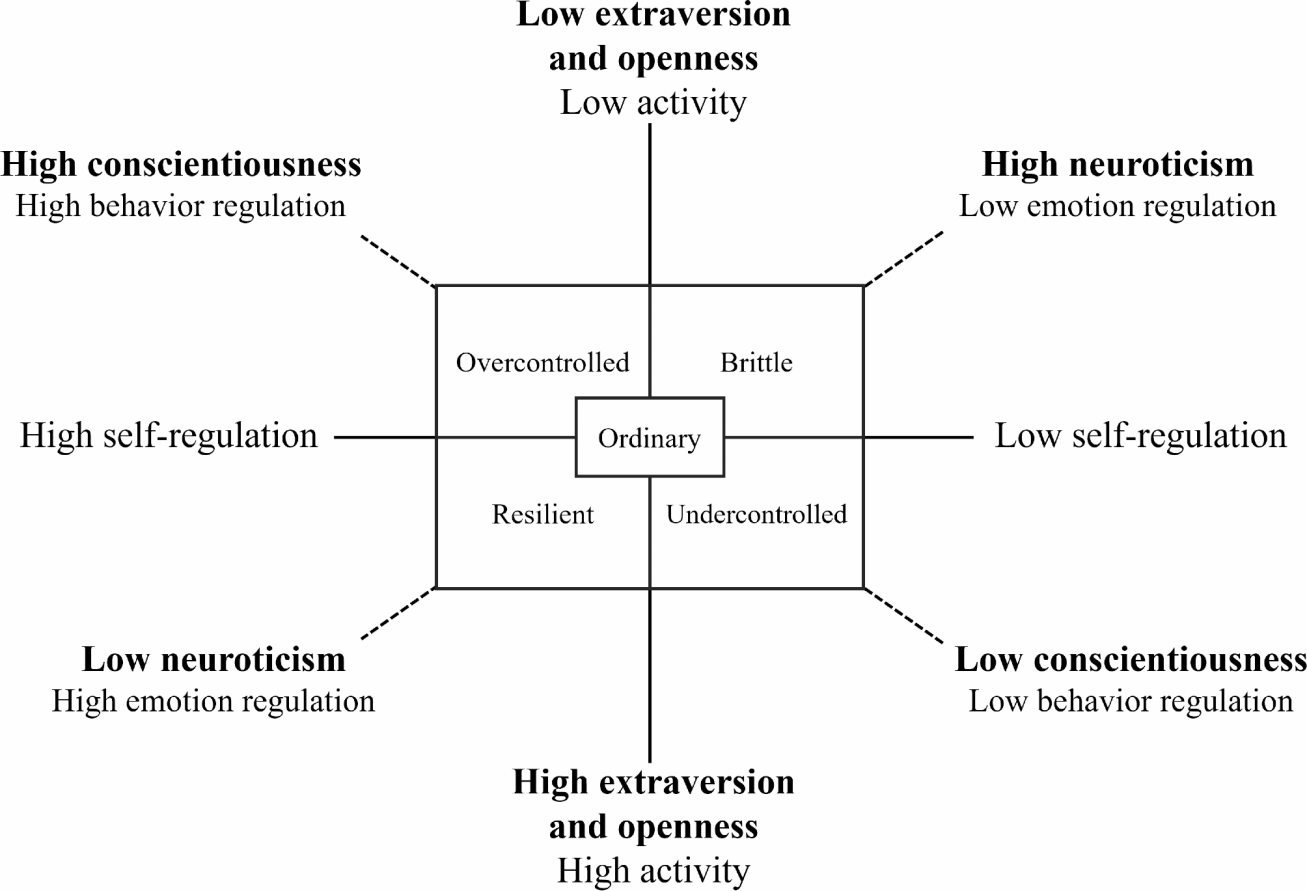
Personality profiles defined by the dimensions of activity and self-regulation. Modified from Figure 4.1 on personality profiles within the framework of activity and self-regulation and Figure 5.4. on personality profiles within the framework of extraversion and conscientiousness by Pulkkinen [17].

There were differences between the profiles within a two-dimensional framework defined by activity (extraversion [E] and openness [O]) and self-regulation—the latter containing two components of emotion regulation (neuroticism [N]) and behavior regulation (conscientiousness [C]) [17] (Fig. 2). Individuals with a *resilient* profile were high in activity (high in E and O) and high in self-regulation, particularly emotion regulation (low in N), whereas those with a *brittle* profile were the opposite (low in E and high in N). Individuals with an *overcontrolled* profile were, in turn, low in activity (low in E and O) and high in self-regulation, particularly behavior regulation (high in C), whereas those with an *undercontrolled* profile were the opposite (high in E and O and low in C). The *ordinary* profile was average in both dimensions.

The selection for providing valid accelerometer data was analyzed by comparing the participants and non-participants altogether and separately in each personality profile (Additional File 1, Table S2). The participation rates according to personality profile were as follows: *resilient* (60%), *brittle* (35%), *overcontrolled* (33%), *undercontrolled* (43%), and *ordinary* (47%). In terms of personality traits, the participants were less neurotic and more agreeable, open, and conscientious than the non-participants. The participation rate was higher among women (55%) than among men (38%). The participants had more commonly higher educational (vocational college, polytechnic, or university) and occupational backgrounds (white collar) rather than lower educational (vocational school at most) and occupational backgrounds (blue collar). The participants also rated their health more often as good. The profile-specific comparisons are presented in Supplementary Material (Additional File 1, Table S2).

On the basis of the chi-square test results for the final sample of 141 participants, educational status [*χ*^2^(4) = 20.209, *p* < 0.001] and self-rated health [*χ*^2^(4) = 10.699, *p* = 0.030], but not gender [*χ*^2^(4) = 5.623, *p* = 0.229] and occupational status [*χ*^2^(4) = 7.712, *p* = 0.103], were statistically significantly associated with the personality profiles. However, according to adjusted standardized residuals, those who had a higher educational degree and good self-rated health were overrepresented in the *resilient* profile (adjusted standardized residuals = 2.6; 2.6). By contrast, those who had not completed higher education and were blue-collar workers were overrepresented in the *brittle* profile (2.1; 2.3). Men were overrepresented in the *overcontrolled* profile (2.3), and those with higher education were overrepresented in the *undercontrolled* (2.7) and underrepresented in the *ordinary* profiles (–2.9).

### Ratios of MVPA to SB by personality profiles

**Fig. 3:**
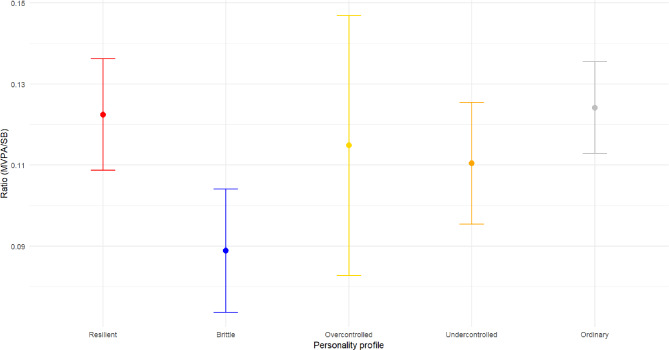
Ratios of MVPA to SB per personality profile (standard error given as the error bar)

There were no statistically significant differences between the personality profiles in their ratios of MVPA to SB [*H*(4) = 2.164, *p* = 0.706] (Fig. 3). However, the individuals with *resilient* and *ordinary* profiles had ratios of 0.12, which corresponded to 7 min of MVPA per hour of daily SB. In contrast, those with a *brittle* profile had a ratio of 0.09, corresponding to 5.5 min of MVPA per hour of daily SB.

### Accumulation patterns of MVPA and SB by personality profiles

Although no statistically significant differences in the daily amount of MVPA was found between the personality profiles [*H*(4) = 3.622, *p* = 0.460], those with a *resilient* profile accumulated the most MVPA per day, and those with a *brittle* profile accumulated the least MVPA per day (Table 2). The differences in the daily amount of SB were not statistically significant [*H*(4) = 1.198, *p* = 0.878].

**Table 2:** Descriptive statistics of the multiple metrics of MVPA and SB (*n* = 141)

		Resilient		Brittle		Overcontrolled		Undercontrolled		Ordinary	
		*n* = 37		*n* = 15		*n* = 10		*n* = 20		*n* = 59	
		M	SD	M	SD	M	SD	M	SD	M	SD
MVPA											
	Daily amount (min/d)	61.8	35.6	41.7	23.5	49.8	34.6	53.7	28.2	56.1	30.7
	Usual bout duration (min)	8.1	10.6	7.2	9.8	2.4	0.8	3.1	2.2	5.4	7.2
	Number of bouts (n/d)	22.8	10.7	17.1	8.0	23.4	16.0	24.9	10.1	24.3	13.1
SB											
	Daily amount (min/d)	531.7	95.8	504.8	91.5	539.5	153.6	516.9	79.5	505.5	106.4
	Usual bout duration (min)	28.8	14.9	21.8	8.9	23.0	11.3	24.0	10.4	22.7	11.8
	Number of bouts (n/d)	61.8	16.5	70.1	13.0	66.3	11.3	64.8	14.5	69.8	14.0

Note. MVPA = moderate-to-vigorous physical activity, SB = sedentary behavior, M = mean, SD = standard deviation

In terms of pattern of accumulation, the individuals with a *resilient* profile exhibited the longest usual MVPA bout duration with respect to the other profiles. Although the main effect was not quite statistically significant [*H*(4) = 8.000, *p* = 0.092], the pairwise comparisons revealed a statistically significant difference between the individuals with *resilient* and *overcontrolled* profiles (*p* = 0.032), and those with *resilient* and *undercontrolled* profiles (*p* = 0.019). Among the single traits, neuroticism (*r = −* 0.17, *p* = 0.043) correlated statistically significantly with usual MVPA bout duration, while other traits of extraversion (*r =* 0.14, *p* = 0.097), openness (*r =* 0.09, *p* = 0.297), agreeableness (*r =* 0.16, *p* = 0.056), and conscientiousness (*r =* 0.13, *p* = 0.117) did not (Additional File 2, Table S5).

The individuals with a *resilient* profile exhibited the longest usual SB bout duration, while those with *brittle* and *ordinary* profiles exhibited the shortest. Although the main effect was not statistically significant [*H*(4) = 6.035, *p* = 0.197], a statistically significant difference was found between the individuals with *resilient* and *ordinary* profiles in the pairwise comparisons (*p* = 0.018). Among the single traits, neuroticism (*r = −* 0.17, *p* = 0.042) correlated statistically significantly with usual SB bout duration, while extraversion (*r =* 0.02, *p* = 0.789), openness (*r =* 0.13, *p* = 0.115), agreeableness (*r =* 0.01, *p* = 0.928), and conscientiousness (*r =* 0.03, *p* = 0.691) did not (Additional File 2, Table S5).

A more detailed description of the accumulation patterns of MVPA and SB pooled according to personality profile is found in the bout distribution plots (Additional File 2, Figure S1, Figure S2). The profiles followed relatively similar accumulation patterns of MVPA and SB, with more variability in the probabilities of longer bouts.

## Discussion

The aim of this study was to identify adults’ personality profiles based on the NEO-FFI and to characterize and investigate how these profiles differ in physical behavior. Five trait combinations, namely *resilient*, *brittle*, *overcontrolled*, *undercontrolled*, and *ordinary*, were identified at ages 33–61 years, similar to those previously reported in the same longitudinal study that covered three earlier measurement points (ages 33, 42, and 50 years) [20]. The profiles differed in the accumulation patterns of MVPA and SB at age 61.

The individuals with *resilient* and *brittle* profiles differed in the dimensions of activity and emotional self-regulation. When looking solely at the ratios of MVPA to SB, the individuals with *resilient* and *ordinary* profiles spent most time in MVPA relative to SB. The findings on the *resilient* profile align with the observations by Nelson [21]. The ratios of these groups, which exceeded 0.10, have been associated with decreased mortality risk [2]. Those with a *resilient* profile also displayed the most prolonged bout patterns of all profiles in terms of MVPA and SB. The findings may relate to the personality traits of higher conscientiousness (e.g., tendency to be organized and hardworking), higher extraversion (e.g., tendency to be active and social), and lower neuroticism (e.g., tendency to be calm and resilient to stress), which have previously been found to be associated especially with greater MVPA levels than those with opposite scores [9–12]. In terms of physical behavior, it may be that compared with those with the other profiles, *resilient* individuals with high scores in conscientiousness have more intentions to engage in PA [42], prefer scheduled activities [43], and set more exercise-related goals [44] that they achieve. Their high levels of extraversion may also be reflected as motivation to exercise for enjoyment; and low levels of neuroticism, as experiencing fewer PA-related barriers such as amotivation and fear of embarrassment [43]. Mechanisms explaining why the *resilient* individuals spent the longest uninterrupted sedentary periods need further investigation. However, the links may be very complex and relate, for example, to them balancing between scheduled exercise and rest, and engaging in sedentary leisure activities such as social media use [13] and work-life demands (e.g., more occupational sitting), as the monitor was worn during the whole day.

Based solely on the ratios of MVPA to SB, the individuals with a *brittle* profile engaged the least in MVPA relative to SB. Their ratio remained under 0.10, which has been associated with increased mortality risk [2]. Since the profile captured characteristics contrary to those of the *resilient* profile in terms of personality traits, the mechanisms may be similar to those mentioned as relating to PA intentions [42], goals [44], preferences, motives, and barriers [43]. However, together with the individuals with an *ordinary* profile, they interrupted their SB most frequently. Individuals with high neuroticism have a tendency to feel nervous and agitated [15] and cope poorly with stress [45], which may be reflected in their physical behavior such that they feel unease to stay sedentary for long periods, and engage in light activities. The links may also pertain to the job characteristics of blue-collar workers, who were overrepresented in the *brittle* group and who are suggested to have less occupational sitting and total SB than those with a higher occupational status [46].

The *overcontrolled* and *undercontrolled* profiles differed in the dimensions of activity and behavioral self-regulation, but the individuals with both profiles engaged in the shortest usual MVPA bout durations. Similar kinds of behavior may be explained by different reasons. The low extraversion of the *overcontrolled* individuals may lead them not to seek sensory-stimulating PA [43]. The profile reflects constrained behavior without neurotic tendencies [17]. Although high scores of conscientiousness could have portended that these self-disciplined individuals with health- rather than appearance-related motives to PA [43] are adherent to the behavior, this was not the case in the present study. The results of the *undercontrolled* individuals also warrant further research but may be related to their tendency to be curious, seek out new experiences [15], prefer spontaneous rather than scheduled exercise [43], and be prone to distractions in tasks that require perseverance [45], which are reflected as fragmented MVPA patterns.

The personality profiles differed modestly in physical behavior, and variation within the profiles was high. Outliers within the personality profiles were likely to contribute to differences in means. Particularly in the *brittle* group, which consisted of only 15 individuals, the outliers with low scores in the usual SB bout duration might have affected the results and led to differences between absolute and rank-order values in comparison with those in the *ordinary* profile. Considering the variability, it is possible that the personality profiles are not clear contributors to accelerometer-measured physical behavior.

In the study of Kinnunen et al. [20], the profiles were associated with subjective (e.g., self-rated health) but not with objective health (e.g., BMI and blood pressure), which may also be the case with complex device-based physical behavior. The individuals with different personality profiles might also have different PA preferences [43], some of which were not captured by the accelerometers. For instance, the individuals who scored high in openness (characteristics of *undercontrolled* profile) are suggested to prefer gym training [43], which is underestimated by the used monitor [33]. Compared with the results of studies that assessed the role of single personality traits in physical behavior, the results of the present study are complicated to interpret, as the personality profiles simultaneously considered all five traits, which may also modify each other’s positive and negative associations with MVPA and SB.

The strength of this study lies in the data used for personality profiling, which were obtained from a representative sample of Finnish adults born in 1959; its ability to mitigate personality trait overlap using a statistical approach; and its capacity to categorize individuals according to their personality data collected at multiple measurement points. Furthermore, this study offers a unique combination of a person-centered approach to personality and novel device-based metrics of physical behavior, which has not been used in studies on personality-PA linkages. Accelerometers are useful in measuring incidental movements of different durations [23] and estimating SB [24], both of which are difficult to capture with self-report owing to the wide variety of particular activities one would have to be prompted on and being less memorable than purposeful activities such as going for a walk or reclining while reading a book. The present study was also assumed to consider potential personality-related differences in reporting PA, as individuals high in neuroticism may report themselves as having less PA than shown by their accelerometer data [22].

This was an indicative study with some limitations. First, there was some attrition among those who participated in the accelerometer measurement in terms of personality profiles and traits, and sociodemographic and health-related characteristics, which shows that attrition in the accelerometer measurement was not fully random. Differences in physical behavior between the personality profiles might have been more evident if there were more participants who scored higher in neuroticism and lower in openness, agreeableness, and conscientiousness. For instance, the individuals in the *brittle* group, who accumulated the least MVPA relative to SB and engaged in the shortest usual SB bout duration among all profiles, might have shown more distinct differences to those with a *resilient* profile without the attrition. Second, a limited number of participants heightened the susceptibility to type II errors, diminishing the likelihood of detecting statistically significant differences. Third, although the high average latent class probabilities for most likely latent class membership suggested that the personality profiles have good classification quality, treating class memberships as known ignored the fact that individuals do not always belong to a particular group with 100% certainty, which might have biased the results. Fourth, during the analysis of the accelerometer data, epochs of 5 s were averaged to 60 s, which, however, might have led to the ignorance of very short bouts of MVPA and SB. The accelerometer measurement was also likely to underestimate non-step-based activities such as cycling, upper-body gym training, and swimming [33]. Fifth, the possibility of reverse causality could not be eliminated owing to the dynamic nature of the variables and the methods used in the present study. Sixth, this study focused on only one layer of personality [8] and did not consider, for example, motivational factors linked to both personality traits and MVPA [47]. Lastly, the data were based on a cohort of Finns, for which the results are generalizable beyond this population with caution. For instance, in other countries, expectations about the role of women may affect their ability to be physically active according to their preferences.

## Conclusions

The novelty of the present study relates to combining a person-centered approach to personality traits with device-based measures of the temporal structure of physical behavior. We identified five distinct personality profiles, which differed in the patterns of accumulation of MVPA and SB. Accruing most MVPA relative to SB—alongside those with an *ordinary* profile—and accumulating MVPA and SB in longer bouts were characteristics of the individuals with a *resilient* profile. By contrast, the individuals with a *brittle* profile engaged in the least MVPA relative to SB but exhibited shorter SB bouts, along with the individuals in the *ordinary* profile. As it is possible that one group of people engage simultaneously in most and longer bouts of MVPA and SB while the other group accumulate least MVPA but interrupt SB more frequently, it is worth examining multidimensional physical behavior by paying attention to both MVPA and SB, and to their amount and accumulation patterns.

The results are indicative and provide justification for further research regarding whether and why personality characteristics are linked to the accumulation of physical behavior. The question of whether the associations between personality profiles and physical behavior differ between men and women remains an interesting, yet unexplored, research topic. The results of this study may facilitate future studies, understanding physical behavior, and intervention planning targeting people with specific personality profiles.

## Electronic supplementary material

Below is the link to the electronic supplementary material.

Supplementary Material 1

Supplementary Material 2

Supplementary Material 3

## Data Availability

Owing to the sensitivity of the data and privacy of the participants, the law dictates that the data cannot be openly shared. Except for the most recent, age 61 data, the data are stored in the Finnish Social Science Data Archive (FSD) (https://www.fsd.uta.fi/en/). The data analyses that support the findings of the present article are available from the corresponding author upon reasonable request. Pseudonymized datasets are available to external collaborators upon agreement on the terms of data use and publication of results. For further information about the data request, please see the Kokko et al. [26] article.

## Abbreviations

MVPA: Moderate-to-vigorous physical activity
PA: Physical activity
SB: Sedentary behavior
