# Supplementary material for: Do personality profiles contribute to patterns of physical activity and sedentary behavior in adulthood? A prospective cohort study
Source: Int J Behav Nutr Phys Act. 2024 Sep 26;21:107. doi: 10.1186/s12966-024-01662-y (PMC11426097; doi:10.1186/s12966-024-01662-y)
Supplement: Supplementary file 1 — Supplementary Material 1 [file 12966_2024_1662_MOESM1_ESM.docx]

**Do personality profiles contribute to patterns of physical activity and sedentary behavior in adulthood? A prospective cohort study**

**Additional File 1**

**Recruitment**

The participants were recruited in 1968 through a random selection of 12 complete second grade school classes in Jyväskylä, Central Finland [1].

**Representativeness of the sample**

The recruitment process resulted in an initial sample of 369 participants without attrition [1,2]. The sample included 53% males. The participants were native Finns born mainly in 1959.

Since the initial data collection, the same participants were followed in major data collection waves [1,2]. Loss to follow-up was addressed by providing flexible options to participate in the data collection, and the reasons behind non-participation at the different measurement points included lack of consent, being deceased, or having unknown contact information. The remaining study sample in adulthood has reasonably well represented both the initial sample and the same-age Finnish cohort on several behavioral and sociodemographic characteristics, respectively [1,2]. More detailed information on the representativeness of the sample can be found in previous publications [1,2].

In the present study, the eligible sample (i.e., participants who have neither deceased nor declined their study participation, and whose contact information is known) and effective sample (i.e., participants who participated in the respective data collection phase) per main measure at each time point (ages 33, 43, 50, and 61 years) are presented in Table S1. The participation percentage ranged from 57.5 to 67.9 for personality and was 79.3 for physical behavior. Among the 179 participants for whom the accelerometer measuring physical behavior was offered, 30 did not want to participate and 7 either suspended the participation or did not wear the monitor for at least 4 days of 10 hours [3].

**Table S1**. Eligible and effective samples of the present analyses for each main outcome measure at each time point.

|  |  | Age 33 | Age 42 | Age 50 | Age 61 |
| --- | --- | --- | --- | --- | --- |
|  |  | n (%) | n (%) | n (%) | n (%) |
| Personality traits | |  |  |  |  |
|  | Eligible sample | 369 | 343 | 323 | 301 |
|  | Non-participants | 120 (32.5) | 110 (32.1) | 111 (34.4) | 128 (42.5) |
|  | Effective sample | 249 (67.5) | 233 (67.9) | 212 (65.6) | 173 (57.5) |
| Physical behavior | |  |  |  |  |
|  | Eligible sample^a^ |  |  |  | 179 |
|  | Non-participants^b^ |  |  |  | 37 (20.7) |
|  | Effective sample |  |  |  | 142 (79.3) |
| ^a^ including those interested in health examination ^b^ also including those not providing valid data | | | | | |

The analytical sample in this study consisted of 141–307 participants, depending on the analysis in question. The latent profile analysis on personality traits included a sample of 307 participants who provided data on personality traits at least once (ages 33, 42, 50 and/or 61 years). Among the 307 participants, 46% (n = 141) provided valid accelerometer data at age 61 years.

The comparisons between those who provided valid accelerometer data (n = 141) and those who did not provide valid accelerometer data (n = 166) among the sample of 307 participants are presented in Table S2. Those with valid accelerometer data differed from the rest of the participants in terms of their personality traits and sociodemographic and health-related characteristics.

**Table S2.** Comparison of attrition between the participants with valid accelerometer data and the non-participants.

|  | | *All (n = 307)* | |  | *Resilient (n = 62)* | |  | *Brittle (n = 43)* | |  | *Overcontrolled (n = 30)* | |  | *Undercontrolled (n = 47)* | |  | *Ordinary (n = 125)* | |  |
| --- | --- | --- | --- | --- | --- | --- | --- | --- | --- | --- | --- | --- | --- | --- | --- | --- | --- | --- | --- |
|  | | Participants (n = 141, 46%) | Non-participants (n = 166, 54%) |  | Participants (n = 37, 60%) | Non-participants (n = 25, 40%) |  | Participants (n = 15, 35%) | Non-participants (n = 28, 65%) |  | Participants (n = 10, 33%) | Non-participants (n = 20, 67%) |  | Participants (n = 20, 43%) | Non-participants (n = 27, 57%) |  | Participants (n = 59, 47%) | Non-participants (n = 66, 53%) |  |
|  | | n (%) | n (%) | p^a^ | n (%) | n (%) | p^a^ | n (%) | n (%) | p^a^ | n (%) | n (%) | p^a^ | n (%) | n (%) | p^a^ | n (%) | n (%) | p^a^ |
| Gender | |  |  | **0.005** |  |  |  |  |  |  |  |  |  |  |  |  |  |  |  |
|  | Women | 78 (54.5) | 65 (45.5) |  | 22 (68.8) | 10 (31.3) | 0.133 | 9 (47.4) | 10 (52.6) | 0.126 | 2 (40.0) | 3 (60.0) | 0.729 | 12 (52.2) | 11 (47.8) | 0.192 | 33 (51.6) | 31 (48.4) | 0.317 |
|  | Men | 63 (38.4) | 101 (61.6) |  | 15 (50.0) | 15 (50.0) |  | 6 (25.0) | 18 (75.0) |  | 8 (32.0) | 17 (68.0) |  | 8 (33.3) | 16 (66.7) |  | 26 (42.6) | 35 (57.4) |  |
| Educational status | |  |  | **0.001** |  |  |  |  |  |  |  |  |  |  |  |  |  |  |  |
|  | Vocational school at most | 68 (38.6) | 108 (61.4) |  | 11 (47.8) | 12 (52.2) | 0.144 | 11 (31.4) | 24 (68.6) | 0.320 | 5 (27.8) | 13 (72.2) | 0.429 | 4 (19.0) | 17 (81.0) | **0.003** | 37 (46.8) | 42 (53.2) | 0.648 |
|  | Vocational college or polytechnic, university | 73 (57.0) | 55 (43.0) |  | 26 (66.7) | 13 (33.3) |  | 4 (50.0) | 4 (50.0) |  | 5 (41.7) | 7 (58.3) |  | 16 (61.5) | 10 (38.5) |  | 22 (51.2) | 21 (48.8) |  |
| Occupational status | |  |  | **0.001** |  |  |  |  |  |  |  |  |  |  |  |  |  |  |  |
|  | Blue-collar worker | 33 (33.7) | 65 (66.3) |  | 5 (41.7) | 7 (58.3) | 0.133 | 7 (25.0) | 21 (75.0) | 0.063 | 3 (25.0) | 9 (75.0) | 0.429 | 3 (27.3) | 8 (72.7) | 0.187 | 15 (42.9) | 20 (57.1) | 0.407 |
|  | White-collar worker | 108 (53.5) | 94 (46.5) |  | 32 (65.3) | 17 (34.7) |  | 8 (53.3) | 7 (46.7) |  | 7 (38.9) | 11 (61.1) |  | 17 (50.0) | 17 (50.0) |  | 44 (51.2) | 42 (48.8) |  |
| Self-rated health | |  |  | **0.013** |  |  |  |  |  |  |  |  |  |  |  |  |  |  |  |
|  | Fairly or very good | 103 (52.8) | 92 (47.2) |  | 33 (64.7) | 18 (35.3) | 0.144 | 8 (44.4) | 10 (55.6) | 0.307 | 5 (27.8) | 13 (72.2) | 0.429 | 15 (50.0) | 15 (50.0) | 0.375 | 42 (53.8) | 36 (46.2) | 0.199 |
|  | Average, fairly or extremely poor | 38 (37.6) | 63 (62.4) |  | 4 (40.0) | 6 (60.0) |  | 7 (29.2) | 17 (70.8) |  | 5 (41.7) | 7 (58.3) |  | 5 (35.7) | 9 (64.3) |  | 17 (41.5) | 24 (58.5) |  |
|  |  | M (SD) | M (SD) | p^b^ | M (SD) | M (SD) | p^b^ | M (SD) | M (SD) | p^b^ | M (SD) | M (SD) | p^b^ | M (SD) | M (SD) | p^b^ | M (SD) | M (SD) | p^b^ |
| Personality traits | |  |  |  |  |  |  |  |  |  |  |  |  |  |  |  |  |  |  |
|  | Neuroticism | 2.15 (0.60) | 2.50 (0.70) | **<0.001** | 1.65 (0.37) | 1.88 (0.50) | 0.054 | 3.26 (0.45) | 3.42 (0.57) | 0.348 | 2.18 (0.50) | 2.20 (0.35) | 0.894 | 2.27 (0.41) | 2.62 (0.63) | **0.036** | 2.14 (0.38) | 2.39 (0.52) | **0.002** |
|  | Extraversion | 3.07 (0.54) | 3.09 (0.59) | 0.792 | 3.53 (0.45) | 3.67 (0.43) | 0.225 | 2.32 (0.37) | 2.51 (0.38) | 0.121 | 2.58 (0.18) | 2.68 (0.50) | 0.476 | 3.06 (0.41) | 3.26 (0.57) | 0.155 | 3.06 (0.41) | 3.16 (0.45) | 0.183 |
|  | Openness | 3.32 (0.50) | 3.15 (0.63) | **0.012** | 3.73 (0.34) | 3.63 (0.38) | 0.272 | 2.97 (0.37) | 2.98 (0.43) | 0.925 | 2.58 (0.33) | 2.24 (0.40) | **0.029** | 3.70 (0.39) | 3.94 (0.41) | **0.044** | 3.14 (0.33) | 3.00 (0.34) | **0.024** |
|  | Agreeableness | 3.93 (0.44) | 3.56 (0.53) | **<0.001** | 4.25 (0.30) | 3.83 (0.42) | **<0.001** | 3.52 (0.43) | 3.37 (0.30) | 0.184 | 3.29 (0.37) | 3.22 (0.61) | 0.770 | 3.76 (0.37) | 3.33 (0.67) | **0.012** | 4.00 (0.32) | 3.73 (0.44) | **<0.001** |
|  | Conscientiousness | 3.76 (0.46) | 3.61 (0.56) | **0.013** | 4.03 (0.35) | 4.04 (0.38) | 0.923 | 3.78 (0.50) | 3.44 (0.54) | **0.048** | 4.14 (0.27) | 3.67 (0.46) | **0.006** | 3.31 (0.39) | 3.08 (0.50) | 0.102 | 3.68 (0.41) | 3.72 (0.49) | 0.544 |
| *Note*. Values based on the participant’s most recent scores. M = mean, SD = standard deviation, p^a^ = p-value for chi-square test, p^b^ = p-value for independent samples t-test. | | | | | | | | | | | | | | | | | | | |

**Missing values**

In the latent profile analysis of the personality traits, the method (full information maximum likelihood with robust standard errors, MLR) utilized information from all available data points. Thus, everyone who provided data on personality traits at least once was included in the analysis (n = 307).

In the attrition analyses, the missing information in background variables inquired at age 61 years was imputed using the participants' latest scores. Among the sample of 307 participants, data on educational status were imputed for 101 participants; occupational status, for 98 participants; self-rated health, for 93 participants; and personality traits, for 134 participants. After the imputation, there was still missing information for 3, 7, 11, and 0 participants, respectively.

In the main analysis, missing accelerometer data were not imputed for the measurement being conducted for the first time at age 61 years.

**References**

1. Pulkkinen L. Human development from middle childhood to middle adulthood: growing up to be middle-aged [In collaboration with Katja Kokko]. London: Routledge. Open access: https://doi.org/10.4324/9781315732947; 2017.

2. Kokko K, Fadjukoff P, Reinilä E, Ahola J, Kinnunen M-L, Kroger J, et al. Developmental Perspectives on Transitions at Age 60: Individuals Navigating Across the Lifespan (TRAILS) – latest data collection in a longitudinal JYLS study. Longitud Life Course Stud. 2024;1–31.

3. Migueles JH, Cadenas-Sanchez C, Ekelund U, Delisle Nyström C, Mora-Gonzalez J, Löf M, et al. Accelerometer data collection and processing criteria to assess physical activity and other outcomes: A systematic review and practical considerations. Sports Med. 2017;47:1821–45.
