# Supplementary material for: Do personality profiles contribute to patterns of physical activity and sedentary behavior in adulthood? A prospective cohort study
Source: Int J Behav Nutr Phys Act. 2024 Sep 26;21:107. doi: 10.1186/s12966-024-01662-y (PMC11426097; doi:10.1186/s12966-024-01662-y)

**Do personality profiles contribute to patterns of physical activity and sedentary behavior in adulthood? A prospective cohort study**

**Additional File 2**

**Table S3.** Goodness-of-fit indices for latent profile analysis (LPA) of personality profiles at ages 33–61.

| No of profiles | BIC | SABIC | AIC | Log-likelihood: H0 Value | Log-likelihood: H0 Scaling Correction Factor for MLR | Entropy | Profile sizes based on their most likely latent class membership | Profile sizes based on estimated posterior probabilities |  |
| --- | --- | --- | --- | --- | --- | --- | --- | --- | --- |
| 1 | 7384.2 | 7257.4 | 7235.2 | –3577.6 | 1.0213 |  | 307 | 307.0 |  |
| 2 | 6842.1 | 6648.6 | 6614.8 | –3246.4 | 1.2011 | 0.764 | 121/186 | 119.4/187.6 |  |
| 3 | 6679.1 | 6419.0 | 6373.5 | –3104.7 | 1.1544 | 0.805 | 162/93/52 | 159.1/95.6/52.3 |  |
| 4 | 6568.5 | 6241.8 | 6184.6 | –2989.3 | 1.1672 | 0.802 | 94/75/38/100 | 95.0/75.4/39.6/97.0 |  |
| **5** | **6513.5** | **6120.2** | **6051.4** | **–2901.7** | **1.2608** | **0.821** | **30/43/125/62/47** | **33.0/44.0/117.8/65.9/46.2** |  |
| 6 | 6493.4 | 6033.5 | 5953.0 | –2831.5 | 1.2006 | 0.821 | 39/22/77/32/77/60 | 39.1/22.7/75.6/33.7/76.4/59.5 |  |
| 7 | 6485.2 | 5958.7 | 5866.5 | –2767.3 | 1.1712 | 0.836 | 21/39/108/29/34/50/26 | 25.2/40.0/99.2/31.5/35.7/49.6/25.8 |  |
| 8 | 6481.3 | 5888.2 | 5784.4 | –2705.2 | 1.2069 | 0.830 | 33/36/60/28/12/50/37/51 | 34.0/35.6/59.3/29.1/12.2/49.4/39.1/48.4 |  |
| *Note*. BIC = Bayesian Information Criterion, SABIC = Sample-size adjusted BIC, AIC = Akaike Information Criterion. Lo-Mendell-Rubin likelihood ratio test indicated a better model-fit until two profile solution. Bootstrapped likelihood ratio test indicated a better model-fit until eight profile solution. | | | | | | | | |  |
|  |  |  |  |  |  |  |  |  |  |

**Table S4**. The associations of personality profiles with multiple metrics of physical behavior based on analysis of variance (ANOVA).

|  | | Model 1^a^ | Model 2^b^ | Model 3^c^ |
| --- | --- | --- | --- | --- |
| Ratio of MVPA to SB | | *F*(4, 136) = 0.828, p = 0.509 |  |  |
| MVPA | |  |  |  |
|  | Daily amount (min/d) | *F*(4, 136) = 1.546, p = 0.192 | *F*(4, 136) = 1.440, p = 0.224 | *F*(4, 136) = 1.197, p = 0.315 |
|  | Usual bout duration (min) | *F*(4, 136) = 2.315, p = 0.061, 1 > 3, 4 (p = 0.026; 0.014) | *F*(4, 136) = 2.339, p = 0.058, 1 > 3, 4 (p = 0.024; 0.017) | *F*(4, 136) = 2.042, p = 0.092, 1 > 4 (p = 0.018) |
|  | Number of bouts (n/d) | *F*(4, 136) = 1.405, p = 0.236, 2 < 4, 5 (p = 0.034; 0.033) | F(4, 136) = 1.189, p = 0.319, 2 < 4 (p = 0.044) | *F*(4, 136) = 1.569, p = 0.186, 2 < 4 (p = 0.033) |
| SB | |  |  |  |
|  | Daily amount (min/d) | *F*(4, 136) = 0.547, p = 0.702 | *F*(4, 136) = 0.693, p = 0.598 | *F*(4, 136) = 0.357, p = 0.839 |
|  | Usual bout duration (min) | *F*(4, 136) = 1.684, p = 0.157, 1 > 5 (p = 0.015) | *F*(4, 136) = 1.667, p = 0.161, 1 > 5 (p = 0.019) | *F*(4, 136) = 1.358, p = 0.252 |
|  | Number of bouts (n/d) | *F*(4, 136) = 2.060, p = 0.089, 1 < 5 (p = 0.009) | *F*(4, 136) = 2.691, p = 0.034, 1 < 2, 5 (p = 0.006; 0.012) | *F*(4, 136) = 1.728, p = 0.148, 1 < 2, 5 (p = 0.021; 0.043) |
| *Note*. Ratio of MVPA to SB, MVPA daily amount and number of bouts square rooted, usual MVPA and SB bout durations cube rooted to obtain normal distribution. 1 = resilient, 2 = brittle, 3 = overcontrolled, 4 = undercontrolled, 5 = ordinary. Pairwise comparisons based on LSD-method. | | | | |
| ^a^ Model 1: unadjusted | | | | |
| ^b^ Model 2: adjusted for wear-time | | | | |
| ^c^ Model 3: adjusted for wear-time, gender, educational status, occupational status and self-rated health | | | | |

**Table S5**. Spearman correlations between personality traits and indicators of physical activity and sedentary behavior (n = 141)

|  | 1. | 2. | 3. | 4. | 5. | 6. | 7. | 8. | 9. | 10. | 11. |
| --- | --- | --- | --- | --- | --- | --- | --- | --- | --- | --- | --- |
| 1. MVPA: daily amount | 1 | .48** | .75** | –.46** | –.46** | .23** | –0.01 | 0.15 | 0.04 | 0.09 | –0.05 |
| 2. MVPA: usual bout duration |  | 1 | –0.11 | 0.04 | 0.01 | –0.05 | –.17* | 0.14 | 0.09 | 0.16 | 0.13 |
| 3. MVPA: number of bouts |  |  | 1 | –.59** | –.57** | .32** | 0.09 | 0.13 | –0.05 | 0.04 | –0.12 |
| 4. SB: daily amount |  |  |  | 1 | .68** | –.20* | –0.14 | –0.12 | 0.04 | 0.02 | 0.05 |
| 5. SB: usual bout duration |  |  |  |  | 1 | –.73** | –.17* | 0.02 | 0.13 | 0.01 | 0.03 |
| 6. SB: number of bouts |  |  |  |  |  | 1 | 0.08 | –0.09 | –0.13 | 0.01 | –0.05 |
| 7. Neuroticism |  |  |  |  |  |  | 1 | –.41** | –.34** | –.34** | –.20* |
| 8. Extraversion |  |  |  |  |  |  |  | 1 | .39** | .41** | 0.07 |
| 9. Openness |  |  |  |  |  |  |  |  | 1 | .36** | 0.10 |
| 10. Agreeableness |  |  |  |  |  |  |  |  |  | 1 | .19* |
| 11. Conscientiousness |  |  |  |  |  |  |  |  |  |  | 1 |
| *Note*. MVPA = moderate-to-vigorous physical activity, SB = sedentary behavior. * p < 0.05, ** p < 0.01. Personality traits based on the participant’s latest measurement (ages 33, 42, 50 or 61). | | | | | | | | | | | |

**Figure S1.** Probability distribution of MVPA bouts per bout length.


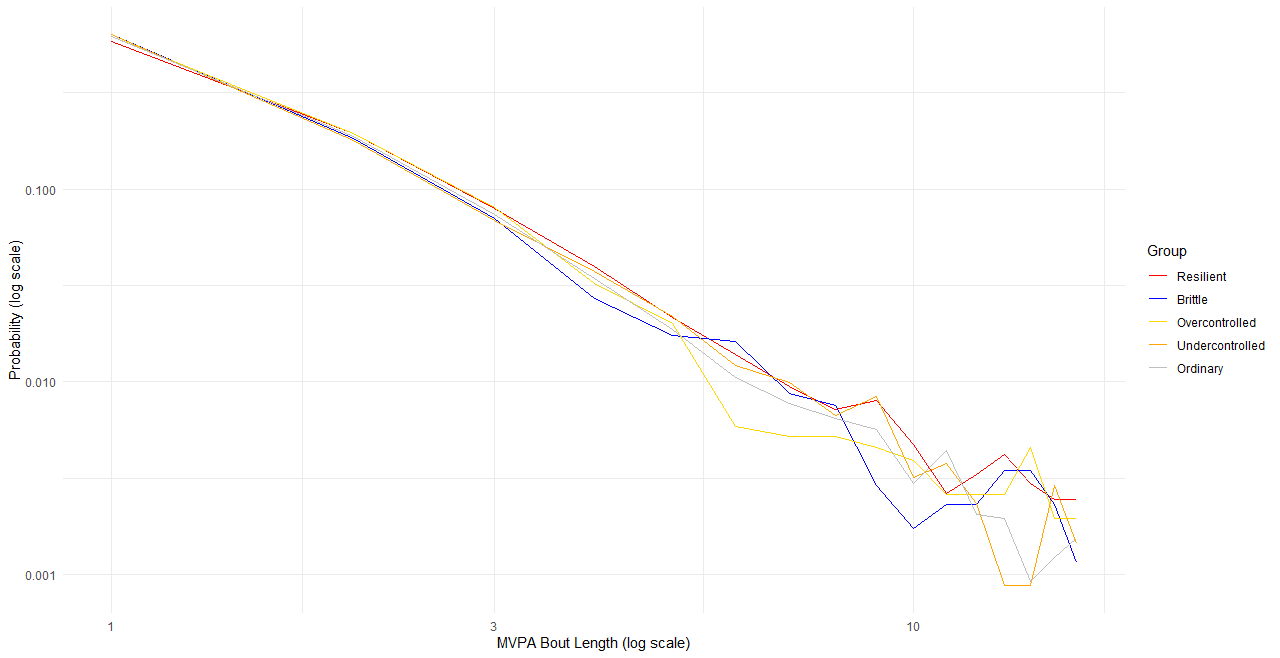


**Figure S2.** Probability distribution of sedentary bouts per bout length.


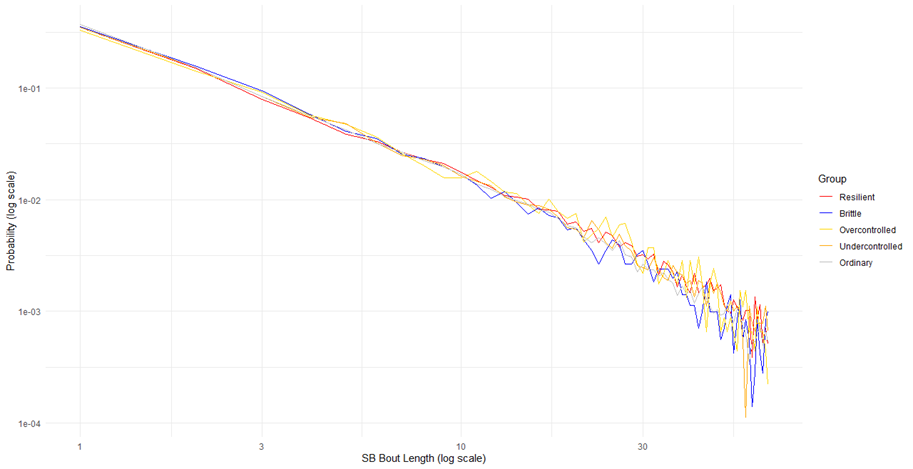

Supplement: Supplementary file 2 — Supplementary Material 2 [file 12966_2024_1662_MOESM2_ESM.docx]
